# Real-world efficacy and safety of CDK4/6 inhibitors plus endocrine therapy in HR+/HER2 − metastatic breast cancer: a single-institution experience

**DOI:** 10.1186/s43046-026-00390-7

**Published:** 2026-07-28

**Authors:** Nawal E. Hussein, Ahmed M. Gad, Abeer AbdAllah, Noha S. El Baghdady

**Affiliations:** 1 Clinical oncology consultant, Head of oncology department, electricity hospital, Cairo, Egypt; 2 Clinical Oncology, Clinical Oncology and Nuclear Medicine Department, Faculty of Medicine, Ain Shams University, Cairo, Egypt; 3 Clinical oncology consultant, Electricity hospital, Cairo, Egypt; 4 Clinical Pharmacy Practice Department, Faculty of Pharmacy, The British University in Egypt, El Sherouk City, Egypt

**Keywords:** CDK4/6 inhibitors, Hormone receptor-positive breast cancer, Endocrine therapy, Real-world evidence

## Abstract

**Background:**

Hormone receptor–positive, HER2-negative (HR+/HER2−) metastatic breast cancer is the most common type of advanced breast cancer. The addition of cyclin-dependent kinase 4/6 (CDK4/6) inhibitors to endocrine therapy (ET) has made a marked change to patient outcomes. While clinical trials have established their efficacy, real-world studies like this one are needed to confirm their effectiveness given the many variables which may arise in patient demographics and clinical presentation.

**Methods:**

A retrospective study was conducted on patients with HR+/HER2 − metastatic breast cancer treated with palbociclib, ribociclib, or abemaciclib plus ET at Electricity Hospital, Cairo, Egypt, between January 2019 and January 2025. Data on demographics, clinical characteristics, and treatment outcomes were collected from medical records. Progression-free survival (PFS) and overall survival (OS) were estimated using the Kaplan–Meier method, and prognostic variables were analyzed using Cox regression.

**Results:**

A total of 59 patients (94.9% female; median age 58 years) were included. De novo metastatic disease was present in 39%, and visceral metastases in 33.9%. Ribociclib was prescribed to 52.5%, palbociclib to 39%, and abemaciclib to 8.5%. The objective response rate (ORR) was 88.1%, and the clinical benefit rate (CBR) was 98.3%. Median PFS was 23.7 months (95% CI, 11.9–23.9), and median OS was 38.9 months (95% CI, 27.0–60.3). On univariate analysis, absence of brain metastases, endocrine sensitivity, treatment response, and neutropenia were linked to longer PFS and OS. Dose reduction did not negatively impact outcomes. Toxicities were generally manageable, with grade 3–4 neutropenia in 33.9% of patients and no febrile episodes.

**Conclusion:**

In this real-world cohort, CDK4/6 inhibitors plus ET achieved survival outcomes similar to those reported in clinical trials, with a favorable safety profile. Endocrine sensitivity, absence of brain metastases, and development of neutropenia were associated with better survival, supporting the use of CDK4/6 inhibitors across a broad patient population.

**Supplementary Information:**

The online version contains supplementary material available at 10.1186/s43046-026-00390-7.

## Introduction

Hormone receptor–positive, human epidermal growth factor receptor 2–negative (HR+/HER2−) breast cancer is the most prevalent molecular subtype of advanced breast cancer, accounting for roughly 70% of cases worldwide [1, 2]. Endocrine therapy (ET) has long been the mainstay of treatment for this subtype; however, the emergence of either primary or acquired endocrine resistance represents a major clinical challenge and is linked to poorer outcomes [2, 3].

The introduction of cyclin-dependent kinase 4/6 (CDK4/6) inhibitors —including palbociclib, ribociclib, and abemaciclib—has markedly changed the therapeutic landscape for HR+/HER2 − metastatic breast cancer. By selectively inhibiting CDK4/6, these agents halt cell cycle progression from the G1 to S phase, thereby enhancing the antitumor activity of endocrine therapy and delaying disease progression. Multiple phase III randomized clinical trials, such as PALOMA [4, 5], MONALEESA [6], and MONARCH [7, 8], have shown significant improvements in both progression-free survival (PFS) and overall survival (OS) when CDK4/6 inhibitors are combined with ET, compared with ET alone. These findings have established CDK4/6 inhibitor-based regimens as standard of care in the first line and subsequent settings [4–8].

While pivotal trials provide strong evidence for efficacy and safety, their patient populations are often highly selected, excluding those with poor performance status, extensive comorbidities, or certain metastatic patterns. Therefore, real-world studies are essential to confirm whether similar outcomes can be achieved in more heterogeneous clinical settings [1, 2, 9]. Furthermore, identifying clinical and treatment-related factors associated with improved outcomes could help refine patient selection and optimize therapeutic strategies [6, 9, 10].

The present study aims to assess the real-world effectiveness and tolerability of CDK4/6 inhibitors combined with endocrine therapy in Egyptian patients with HR+/HER2 − metastatic breast cancer. Additionally, we investigate potential prognostic factors for PFS and OS, with particular attention to endocrine sensitivity, metastatic burden, and treatment-related toxicities.

## Patients and methods

### Study design and population

This retrospective observational study was conducted at the Electricity Hospital, Cairo, Egypt, including patients diagnosed with HR+/HER2 − metastatic breast cancer who received a CDK4/6 inhibitor in combination with endocrine therapy between January 2019 and January 2025. Eligible patients were identified through the hospital medical records system.

Inclusion criteria were:

1. Histologically confirmed HR+/HER2 − breast cancer according to ASCO/CAP guidelines.
2. Presence of metastatic or locally advanced, unresectable disease.
3. Treatment with palbociclib, ribociclib, or abemaciclib in combination with an aromatase inhibitor or fulvestrant.
4. Availability of complete clinical, pathological, and follow-up data.

Patients with missing baseline data or who received CDK4/6 inhibitors in the adjuvant setting only were excluded.

### Data collection

Demographic, clinicopathological, and treatment-related data were collected, including age, sex, menopausal status, HER2 status (negative vs. low expression), disease presentation (de novo vs. recurrent), metastatic sites (bone, liver, lung, brain), visceral involvement, endocrine sensitivity (sensitive, primary resistant, secondary resistant), CDK4/6 inhibitor type, treatment line, endocrine partner, and use of ovarian suppression. Toxicity data were graded according to CTCAE v5.0.

### Outcome measures

The primary endpoints were progression-free survival (PFS) and overall survival (OS).

- PFS: time from initiation of CDK4/6 inhibitor until radiologically confirmed progression or death from any cause.
- OS: time from initiation of CDK4/6 inhibitor until death from any cause.

Secondary endpoints included objective response rate (ORR), clinical benefit rate (CBR), and safety profile. Tumor response was evaluated according to RECIST v1.1 criteria [11].

### Statistical analysis

Descriptive statistics were used to summarize the baseline characteristics of the study population. Continuous variables were presented as means ± standard deviation (SD) or medians and interquartile ranges (IQRs), while categorical variables were summarized as frequencies and percentages.

Categorical variables were compared using the Chi-square test or Fisher’s exact test, as appropriate. For continuous variables, normality of the data was assessed using the Shapiro–Wilk test, and Student’s t-test or Mann–Whitney U test was used for inferential analysis, as appropriate.

Survival outcomes were analyzed using the Kaplan–Meier method, median survival times were reported, and differences between groups were assessed using the log-rank test.

Time-to-event outcomes were analyzed using a multivariable Cox proportional hazards regression model. Potential predictors of survival were first evaluated using univariable methods, including Kaplan–Meier survival analysis and the log-rank test to compare survival distributions between groups. Clinically relevant variables and universal confounders, as well as variables with a p-value < 0.10 in the log-rank test, if any, were considered for inclusion in the multivariable Cox regression model. Results were presented as hazard ratios (HRs) with 95% confidence intervals (CIs). A two-sided p-value < 0.05 was considered statistically significant.

Statistical analyses were performed using MedCalc Statistical Software version 23.1.6 (MedCalc Software Ltd, Ostend, Belgium).

## Results

A total of 59 patients (3 males and 56 females) met the eligibility criteria between January 2019 and January 2025. All Baseline demographic, disease, and treatment characteristics were extracted from electronic medical records and are summarized in Table 1.

**Table 1:** Baseline demographic, clinicopathologic, and treatment characteristics

Characteristics	*n* (%)
Age (Y)	
< 40	4 (6.8%)
40–60	33 (55.9%)
> 60	22 (37.3%)
Median age	58 Range (30–77)
Sex	
Male	3 (5.1%)
Female	56 (94.9%)
Menopausal status at start of CDK4/6 inhibitor	
Pre-menopausal	18 (32.1%)
Post-menopausal	38 (67.9%)
Her2/neo status	
Negative	52 (88.1%)
Low	7 (11.9%)
Disease status at Dx.	
de Novo metastases	23 (39.0%)
Recurrent	36 (61.0%)
Visceral metastases	
Yes	20 (33.9%)
No	39 (66.1%)
Site of metastases	
Bone	42 (71.2%)
Lung	12 (20.3%)
Liver	13 (22.0%)
Brain	1 (1.7%)
Endocrine sensitivity	
Sensitive	31 (52.5%)
Resistant	28 (47.5%)
Type of Endocrine Resistance	
Primary	7 (11.9%)
Secondary	21 (35.6%)
CDK4/6 inhibitor Treatment line	
First line	45 (76.3%)
Subsequent line	14 (23.7%)
CDK4/6 inhibitor type	
Palbociclib	23 (39.0%)
Ribociclib	31 (52.5%)
Abemaciclib	5 (8.5%)
Hormonal treatment partner	
AI	40 (67.8%)
Fulvestrant	19 (32.2%)
Goserelin	
Yes	13 (22.0%)
No	46 (78%)

Data are presented as number (%) unless otherwise indicated

*CDK4/6 inhibitor* Cyclin-dependent kinase 4/6 inhibitor, *AI* Aromatase inhibitor, *HER2* Human epidermal growth factor receptor 2

The median age at initiation of CDK4/6 inhibitor plus endocrine therapy was 58 years (range, 30–77 years), about two thirds of the 56 included women were postmenopausal 38 (67.9%), LRHR analogue - goserelin, was administered to 13 (72.2%) premenopausal women. About 40% of our patients had de novo metastatic disease and approximately 60% presented with recurrent disease. Visceral metastases were present in 33.9% of patients, with lung and liver metastases represented around 20% each.

Endocrine resistance was observed in 28 patients (47.5%) with primary resistance accounts for (11.9%, 7 patients), and secondary resistance for about (35.6%, 21 patients).

A CDK4/6 inhibitor was introduced in the first line treatment of more than three quarters (76%) of our patients, while it was introduced in the subsequent treatment (2nd line and beyond) of around one quarter (24%) of our patients.

Ribociclib was the most frequently prescribed CDK4/6 inhibitor, followed by Palbociclib and Abemaciclib (52.5%, 39% and 8.5% respectively), an aromatase inhibitor was the endocrine partner in most patients 67.8%, whereas fulvestrant was used in 32.2% of patients.

The median duration of follow up from starting CDK4/6 inhibitor was 41.3 months (95%CI: 34.4 to 53.1). At the time of data collection, 28 patients 47.5%, were still alive and receiving a CDK4/6 inhibitor /ET. The objective response rate was 88.1% (CR 28.8% and PR 59.3%), only 1.7% had PD, and around 10% of patients had SD, with a CBR exceeding 98% Table 2.

**Table 2:** Objective response according to RECIST version 1.1

Response	*N* (%)
CR	17 (28.8%)
PR	35 (59.3%)
SD	6 (10.2%)
PD	1 (1.7%)
ORR	52 (88.1%)
CBR	58 (98.3%)

*CR * Complete response, *PR* Partial response, *SD* Stable disease, *PD* Progressive disease, *ORR* Objective response rate, *CBR* Clinical benefit rate

**Fig. 1:**
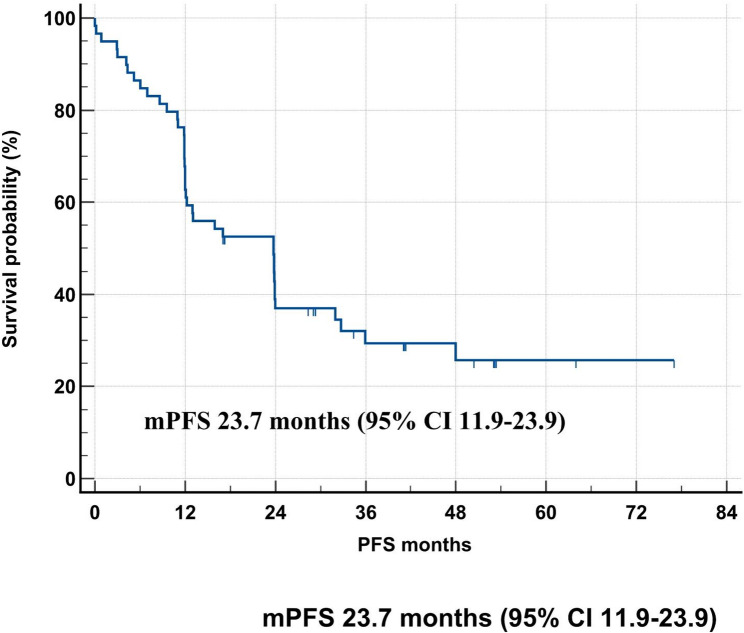
Kaplan–Meier estimates of progression-free survival in the overall study population

**Fig. 2:**
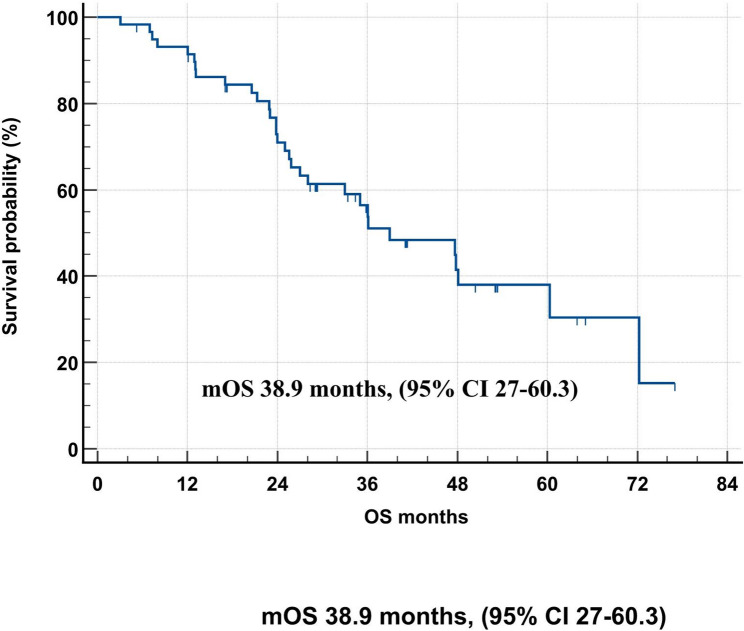
Kaplan–Meier estimates of overall survival in the overall study population

The median progression free survival was 23.7 months (95% CI 11.9–23.9) Fig. 1, and the median overall survival was 38.9 months, (95% CI 27-60.3) for the whole group Fig. 2.

Being free of brain metastases, having an endocrine sensitive disease, Her2 negative versus Her2 low expression tumors, achieving response to treatment, developing neutropenia as a treatment toxicity, and receiving dose reduced CDK4/6 inhibitor are all factors associated with statistically significant impact of better PFS and OS in univariate analysis, Having AI as a hormonal partner was associated with significantly better OS in univariate analysis but not PFS.

The multivariate Cox regression analysis for overall survival included age, gender, type of endocrine sensitivity, CDK4/6 inhibitor type, liver metastasis, endocrine partner, and goserelin use. No individual variable reached statistical significance in the multivariate model; however, several clinically relevant trends were observed. Patients receiving Fulvestrant had a numerically higher risk of death compared to those on aromatase inhibitors (HR 2.10; 95% CI 0.84–5.26, *p* = 0.114), aligning with the trend seen in univariate analysis (*p* = 0.054). Primary endocrine resistance was associated with a higher hazard ratio compared to endocrine-sensitive disease (HR 1.50; 95% CI 0.45–5.04, *p* = 0.510), though not statistically significant. Younger age at diagnosis showed a potential trend toward better survival: patients < 40 years had lower HRs vs. > 60 (HR 0.56; 95% CI 0.04–8.53), though with wide confidence intervals due to small sample size. Use of Abemaciclib was associated with numerically lower hazard for OS compared to Palbociclib (HR 0.27; 95% CI 0.03–2.18), though not statistically significant (*p* = 0.219). goserelin use also showed a trend toward improved OS (HR 0.47; 95% CI 0.06–3.57), again without reaching significance Table S1.

Overall, the multivariate model did not identify statistically significant independent predictors for OS, likely due to limited sample size and event rate. However, the trends observed reinforce findings from prior trials regarding the prognostic role of endocrine sensitivity and CDK4/6 inhibitor -hormonal combinations Table S2.

The most frequently reported adverse events of any grade were neutropenia, anemia, thrombocytopenia, diarrhea, and hepatic toxicity. The most common reported grade 3 or 4 toxicity were neutropenia (33.9%), No cases of febrile neutropenia were reported, or toxicity that mandated hospitalization, G3-4 anemia was the second most common severe adverse event (11.9%), QTC prolongation was only reported in one patient (2%). Detailed toxicity data in Table 3.

**Table 3:** Treatment-related adverse events according to CTCAE version 5.0

Toxicity	*n* (%)
Neutropenia	
Any grade	37 (63.8%)
G3&4	20 (33.9%)
Febrile Neutropenia	Zero
Anemia	
Any grade	28 (47.5%)
G3&4	7 (11.9%)
Thrombocytopenia	
Any grade	1 (1.7%)
G3&4	1 (1.7%)
Diarrhea	
Any grade	3 (5.1%)
G3&4	1 (1.7%)
Vomiting	Zero
Hepatic Toxicity	
Any grade	2 (3.4%)
G3&4	Zero
QTC prolongation	1 (2.0%)

Data are presented as number (%)

## Discussion

This research adds to the real-world evidence on the use of CDK4/6 inhibitors along with endocrine therapy on HR+/HER2 − breast cancer patients with advanced stage disease. The ORR was 88.1% with a CBR of 98.3% which are comparable to results from the PALOMA, MONALEESA, and MONARCH studies [4–8].

The outcomes observed in our cohort were comparable to those reported in pivotal clinical trials despite the inclusion of patients with more diverse clinical characteristics. This consistency is further supported by very recent, large-scale real-world evidence, such as the multicenter study from the Dongting Lake region in China involving 590 patients, which reinforces the role of CDK4/6 inhibitors as a standard treatment across diverse clinical settings [12].

The median PFS was 23.7 months, and the median OS reached 38.9 months, consistent with findings from real-world registries [1, 13, 14] and randomized trials [3–8, 15]. Univariate analysis showed that endocrine sensitivity, absence of brain metastases, treatment response, and neutropenia were significantly associated with longer PFS and OS. These results are in line with previous reports highlighting the prognostic importance of endocrine sensitivity [5, 6, 10, 16], early tumor response [7, 8, 16].

Interestingly, neutropenia has been associated with improved survival outcomes, suggesting it may serve as a pharmacodynamic biomarker of effective CDK4/6 inhibition [9, 13–16]. A novel insight suggests that host inflammatory status may influence treatment outcomes from a recent 2025 study on the neutrophil-lymphocyte ratio (NLR), which found that elevated baseline NLR is associated with both a higher risk of severe neutropenia and poorer survival, suggesting a potential link between the host’s immune-inflammatory state and the efficacy of CDK4/6 inhibitors [17].

Furthermore, dose reduction did not appear to compromise efficacy, consistent with previous analyses from MONALEESA and other studies [16, 18]. Real-world head-to-head comparisons between palbociclib and ribociclib from the OPAL registry further confirm that different CDK4/6 inhibitors offer comparable efficacy in first-line treatment, although ribociclib might show advantages in endocrine-resistant disease [19].

In multivariate analysis, no variable reached statistical significance as an independent predictor of overall survival, most likely due to the limited sample size, which reduces statistical power. Nonetheless, the patterns we observed remain clinically relevant. Patients treated with abemaciclib or with concurrent ovarian suppression showed a numerical trend toward longer survival, in line with results reported in prior studies [7, 8, 15, 16]. On the contrary, outcomes with fulvestrant appeared less favorable compared to aromatase inhibitors, which may reflect its frequent use in patients with heavily pretreated or endocrine-resistant disease [10, 13, 14]. Although these findings did not reach statistical significance, they suggest potentially meaningful differences that warrant further validation in larger, prospective cohorts [13–20].

In terms of safety, our findings were consistent with those reported in pivotal trials and other real-world studies [1, 3–8, 18–14]. Neutropenia was the most frequent adverse event, more commonly observed with palbociclib and ribociclib, but was generally manageable with temporary interruption or dose reduction. Importantly, no febrile neutropenia was seen in our patients [1, 4–6, 9, 15–14]. This aligns with previous reports and supports the observation that hematologic toxicity, although common, is rarely the cause of serious complications in routine practice [9, 13, 14, 18]. Diarrhea was confined to abemaciclib-treated patients, consistent with its known profile [7, 8]. Other events—fatigue, transaminase elevations, and alopecia—were less frequent and rarely exceeded grade 2 [4, 6, 7, 15, 18]. Although QTc prolongation was uncommon in our cohort, A recent systematic review and meta-analysis (2026) on cardiovascular adverse events has further refined our understanding of the safety profiles, confirming that ribociclib is associated with QTc prolongation, while abemaciclib carries a higher risk of venous thromboembolism (VTE); this mandates drug-tailored monitoring rather than a class-wide approach, which is an important consideration for clinical practice [21]. Serious adverse events and treatment discontinuations due to toxicity were uncommon [1, 4, 6, 7, 18–14]. Collectively, these data confirm a favorable tolerability that facilitates sustained therapy in the metastatic setting and may, in turn, support the PFS and OS outcomes observed [5, 13–16, 18].

Taken together, our data confirm that CDK4/6 inhibitors combined with endocrine therapy provide substantial clinical benefit with manageable toxicity in real-world practice. This strengthens the rationale for their use as standard of care in HR+/HER2 − metastatic breast cancer, while also highlighting the need for continued evaluation of biomarkers and real-world predictors of response to optimize treatment selection [13–20].

### Limitations

This study has several limitations, including its retrospective nature, the relatively small sample size, and the limited number of events for OS, which may have underpowered the multivariate analyses. Moreover, biomarker data (e.g., Ki-67, ESR1 mutation) were not routinely available and thus not included in modeling. Despite these limitations, the results provide valuable insights into real-world use of CDK4/6 inhibitors in an underrepresented patient population.

## Conclusion

The combination of CDK4/6 inhibitors with endocrine therapy shows remarkable efficacy and tolerability in real-world practice including patients with endocrine-resistant disease or visceral metastases. Although no independent predictors of survival emerged from multivariate analysis, sensitivity to endocrine therapy, tumor response, and neutropenia remained associated with improved survival outcomes. These findings should be confirmed in larger prospective studies to identify and validate predictive biomarkers for response to CDK4/6 inhibitor therapy.

## Supplementary Information

Supplementary Material 1.

## Data Availability

The datasets created during and/or analysed during the current study are available from the corresponding author on sensible request.
