# Supplementary material for: Real-world efficacy and safety of CDK4/6 inhibitors plus endocrine therapy in HR+/HER2 − metastatic breast cancer: a single-institution experience
Source: J Egypt Natl Canc Inst. 2026 Jul 28;38:52. doi: 10.1186/s43046-026-00390-7 (PMC13415414; doi:10.1186/s43046-026-00390-7)
Supplement: Supplementary file 1 — Supplementary Material 1. [file 43046_2026_390_MOESM1_ESM.docx]

**Table S1. Univariate analysis of progression-free survival and overall survival.**

| Variable | Comparator | Progression Free Survival | | | Overall Survival | | |
| --- | --- | --- | --- | --- | --- | --- | --- |
|  |  | **Median** | **95% CI** | ***P* Value** | **Median** | **95% CI** | ***P* Value** |
| Age | **<40**  **40-60**  **>60** | **23.98**  **23.75**  **17.01** | **5.19-35.9**  **11.99-32.7**  **8.6-31.9** | **0.64** | **72.240**  **47.668**  **32.983** | **25.0 to 48.0**  **23.8 to 60.3** | **0.31** |
| Disease status at Diagnosis | **De Novo Met.**  **Localized** | **13.0**  **23.8** | **11.8-23.9**  **11.9-35.9** | **0.415** | **38.9**  **36.1** | **23.9-60.3**  **23.8-72.2** | **0.58** |
| Visceral met. At start of CDK4/6 inhibitor | **No**  **Yes** | **23.8**  **11.9** | **12.9-31.9**  **4.3-47.9** | **0.286** | **36.1**  **47.6** | **22.5-48.0**  **22.8-60.3** | **0.38** |
| Site of Metastasis | **Liver**  **Yes**  **No** | **11.8**  **23.8** | **2.9-35.9**  **12.9-32.7** | **0.075** | **28.0**  **38.9** | **12.0-47.7**  **27.0-60.3** | **0.13** |
|  | **Lung**  **Yes**  **No** | **11.9**  **23.8** | **4.3-47.9**  **12.2-31.9** | **0.731** | **47.6**  **36.1** | **20.5-60.3**  **27.0-72.2** | **0.89** |
|  | **Bone**  **Yes**  **No** | **17.0**  **23.8** | **11.0-23.8**  **6.7-19.0** | **0.432** | **38.9**  **47.7** | **25.5-60.3**  **22.8-47.7** | **0.57** |
|  | **Brain**  **Yes**  **No** | **4.1**  **23.7** | **12.0-31.9** | **0.0057** | **28.0**  **38.9** | **27.0-60.3** | **0.44** |
| Her2/neo | **Negative**  **Low** | **23.75**  **9.5** | **12.0-31.9**  **0.0-23.8** | **0.417** | **47.6**  **20.5** | **27.0-72.2**  **6.96-36.0** | **0.29** |
| Endocrine sensitivity | **Sensitive**  **1ry Resistance**  **2ry Resistance** | **23.8**  **11.0**  **23.8** | **12.2-47.9**  **0.16-11.9**  **11.8-35.9** | **0.028** | **38.9**  **25.5**  **47.6** | **25.0-60.3**  **22.8-47.7**  **20.5-72.2** | **0.13** |
| CDK4/6 inhibitor treatment line | **First line**  **Subsequent line** | **23.8**  **11.9** | **12.2-32.7**  **9.5-23.8** | **0.369** | **47.6**  **35.0** | **28.0-72.2**  **20.5-47.7** | **0.59** |
| CDK4/6 inhibitor Type | **Palbociclib**  **Ribociclib**  **Abemaciclib** | **23.8**  **15.9** | **11.9-47.9**  **11.8-31.9** | **0.551** | **36.0**  **38.9**  **NR** | **23.8-60.3**  **23.9-72.2** | **0.47** |
| Hormonal treatment partner | **AI**  **Fulvestrant** | **23.8**  **15.9** | **11.9 -47.9**  **11.8-23.8** | **0.187** | **48.0**  **27.0** | **35.0-60.3**  **22.9-47.7** | **0.049** |
| Menopausal status at start of CDK4/6 inhibitor | **Post**  **Pre** | **23.7**  **12.9** | **11.9-32,7**  **11.0-35.9** | **0.764** | **38.9**  **47.6** | **25.5-60.3**  **21.2-72.2** | **0.91** |
| Adding goserelin | **Yes**  **No** |  |  |  | **72.2**  **36.1** | **23.8-72.2**  **25.0-48.0** | **0.10** |
| Objective response | **Response**  **No response** | **23.8**  **11.8** | **2.9-17.0**  **12.0-35.9** | **0.017** | **47.7**  **23.9** | **32.9-72.2**  **3.0-38.9** | **0.03** |
| Neutropenia | **Yes**  **No** |  |  |  | **60.3**  **25.8** | **32.9-72.2**  **21.2-38.9** | **0.009** |
| Grade of neutropenia | **G0**  **G1**  **G2**  **G3** | **13.042**  **4.172**  **31.965**  **23.752** | **11.8-23.8**  **11.8-31.9**  **11.0-35.9** | **0.005** | **25.8**  **28.0**  **60.3** | **21.2-38.9**  **23.8-72.2** | **0.038** |
| Dose reduction | **Yes**  **No** | **47.9**  **12.9** | **12.0-47.9**  **11.8-23.8** | **0.053** | **60.3**  **36.0** | **25.5-47.7** | **0.13** |

PFS, progression-free survival; OS, overall survival; CI, confidence interval. Data are presented as number (%). Survival estimates were calculated using the Kaplan–Meier method and compared using the log-rank test.

**Table S2. Multivariable Cox proportional hazards regression analysis for overall survival.**

| **Characteristic** |  | **All** | **HR (univariable)** | **HR (multivariable)** |
| --- | --- | --- | --- | --- |
| **Age at diagnosis of metastases** | **>60** | **22 (37.3)** | **-** | **-** |
|  | **40-60** | **33 (55.9)** | **0.62 (0.29-1.31, p=0.211)** | **0.60 (0.25-1.45, p=0.258)** |
|  | **<40** | **4 (6.8)** | **0.40 (0.08-1.90, p=0.247)** | **0.56 (0.04-8.53, p=0.676)** |
| **Gender** | **Female** | **56 (94.9)** | **-** | **-** |
|  | **Male** | **3 (5.1)** | **0.75 (0.17-3.33, p=0.704)** | **1.73 (0.15-19.40, p=0.658)** |
| **Type of endocrine sensitivity** | **Sensitive** | **31 (52.5)** | **-** | **-** |
|  | **Primary resistant** | **7 (11.9)** | **2.16 (0.83-5.63, p=0.115)** | **1.50 (0.45-5.04, p=0.510)** |
|  | **Secondary resistant** | **21 (35.6)** | **0.77 (0.33-1.80, p=0.552)** | **0.78 (0.28-2.19, p=0.642)** |
| **Type of CDK4/6 Inhibitor** | **Palbociclib** | **23 (39.0)** | **-** | **-** |
|  | **Abemaciclib** | **5 (8.5)** | **0.31 (0.04-2.33, p=0.253)** | **0.27 (0.03-2.18, p=0.219)** |
|  | **Ribociclib** | **31 (52.5)** | **0.85 (0.41-1.76, p=0.654)** | **0.72 (0.30-1.70, p=0.454)** |
| **liver metastasis** | **no** | **46 (78.0)** | **-** | **-** |
|  | **yes** | **13 (22.0)** | **1.79 (0.82-3.94, p=0.145)** | **1.46 (0.53-4.03, p=0.462)** |
| **Endocrine treatment partner** | **AI** | **40 (67.8)** | **-** | **-** |
|  | **Fulvestrant** | **19 (32.2)** | **2.04 (0.99-4.22, p=0.054)** | **2.10 (0.84-5.26, p=0.114)** |
| **Goserelin** | **No** | **46 (78.0)** | **-** | **-** |
|  | **Yes** | **13 (22.0)** | **0.43 (0.15-1.25, p=0.120)** | **0.47 (0.06-3.57, p=0.467)** |

**HR**, hazard ratio; **CI**, confidence interval; **AI**, aromatase inhibitor. Hazard ratios were estimated using Cox proportional hazards regression analysis. Clinically relevant covariates were included in the multivariable model.
